# Influence of Wearing Corsets During Radiation Therapy in Patients With Thoracic or Lumbar Spinal Bone Metastases

**DOI:** 10.7759/cureus.84093

**Published:** 2025-05-14

**Authors:** Yoshiteru Akezaki, Eiji Nakata, Masato Kikuuchi, Yoshimi Katayama, Haruyoshi Katayama, Takuto Itano, Masanori Hamada, Shinsuke Sugihara

**Affiliations:** 1 Division of Physical Therapy, Kochi Professional University of Rehabilitation, Kochi, JPN; 2 Department of Orthopedic Surgery, Okayama University Hospital, Okayama, JPN; 3 Department of Rehabilitation Medicine, National Hospital Organization Shikoku Cancer Center, Ehime, JPN; 4 Department of Rehabilitation Medicine, Okayama University Hospital, Okayama, JPN

**Keywords:** activities of daily living, corset, pain, radiation therapy, spinal bone metastases

## Abstract

Background

This study aimed to examine the influence of wearing a corset with radiation therapy (RT) on pain, activities of daily living (ADL), and quality of life (QoL) in patients with thoracic or lumbar spinal bone metastases one month after RT.

Methodology

Fifty-two patients (24 males and 28 females) with thoracic or lumbar spinal bone metastases whose measurements were recorded at our institute between July 2012 and December 2016 were included in this study. Age, sex, ADL, pain, spinal instability, and QoL were investigated in our analyses. Patients were divided into stable (0-6 points) and unstable (7-18 points) groups based on their spinal instability neoplastic score. Patients in the stable and unstable groups performed early mobilization depending on their condition. The unstable group wore corsets. The corsets were soft and were worn for three months from the start of RT.

Results

The unstable group showed significant improvements in ADL and QoL and a significant reduction in pain one month after RT (*P* < 0.05). The stable group showed a significant improvement in QoL one month after RT (*P* < 0.05).

Conclusions

Corsets were effective for enabling early movement without lowering QoL in patients with spinal instability of thoracic or lumbar bone metastases.

## Introduction

Bone is a common site of metastasis in patients with advanced cancers. Primary breast, lung, prostate, and kidney cancers frequently metastasize to bone [1-4]. Bones of the spine represent the most common site of skeletal metastasis [5], with 60%-70% of patients with advanced cancers developing spinal metastases [3,6,7,8]. Bone metastases may result in skeletal-related events (SREs), such as pain, spinal cord compression, pathological fractures, and malignant hypercalcemia [9]. Often resulting in considerable pain, SREs impair patient mobility and reduce their quality of life (QoL) [10,11]. Furthermore, SREs are associated with an increased risk of death and increased healthcare costs [12-14].

Radiation therapy (RT) is a common approach for treating cancer patients with bone metastases [15] and can be effective in controlling bone pain with minimal toxicity. Additionally, rehabilitation improves pain and mobility for patients in palliative care with stable vertebral column bone metastases [16]. However, patients with vertebral metastases are in pain and have reduced activity levels. Corsets can fix the spine, reduce pain, and improve QoL. However, few reports have examined the efficacy of using corsets in patients with vertebral metastases.

Therefore, this study aimed to examine whether corsets and RT influence pain, activities of daily living (ADLs), and QoL in patients with thoracic and lumbar bone metastases measured one month after RT.

## Materials and methods

Patients

Fifty-two patients (24 males and 28 females) with thoracic or lumbar spinal bone metastases whose measurements were available from before starting RT to one month after RT at our institution between July 2012 and December 2016 were included in this study. None of the patients underwent surgery, and those who had paralysis were excluded. The median age of the patients was 66 years (range: 40-75 years). The patients had Th10-L5 bone metastases. We conducted this study by the ethical standards of the Shikoku Cancer Center Ethics Committee, which approved this study (Approval No. 114).

Spinal instability assessment

A spinal instability assessment was performed using the spinal instability neoplastic score (SINS) [17]. The SINS is classified into three categories: stable (0-6 points), potentially unstable (7-12 points), and unstable (13-18 points) [18,19]. However, spinal instability was classified into stable (0-6 points) and unstable (7-18 points) groups in this study. At the start of RT, the stable group consisted of 25 patients, and the unstable group had 27 patients.

To prevent disuse syndrome, patients in both the stable and unstable groups performed early mobilization depending on their condition. The unstable group wore corsets. The corset was custom-fit to each patient and worn for three months from the start of RT.

Clinical Parameters

A range of clinical parameters was recorded, including age, sex, pain, ADL, QoL, primary cancer site, spinal bone metastases level, and spinal instability. Pain, ADL, and QoL were measured at the start of RT and one, two, three, four, five, and six months after RT. We defined the assessment window as within ± a few days of each scheduled monthly visit. On the day of the evaluation, the patient was diagnosed by a physician and measured by a physician, physical therapist, or occupational therapist on that day.

Pain assessment: Pain was evaluated using the numeric rating scale (NRS). The NRS was performed with 0 indicating *no pain* and 10 indicating *pain as bad as you can imagine*. Pain was evaluated while standing and without a corset.

ADL assessment: The Barthel index, which can measure ADL independence, was used to assess ADL [20]. The Barthel index evaluates the patient’s ability to perform hair grooming, dressing, feeding, walking, transferring, climbing stairs, using the toilet, and bathing independently. Higher scores indicate a higher degree of ADL independence [21]. Patients’ walking ability was classified into independent walking with 15 points and dependent walking with 0, 5, or 10 points in the mobility items of the Barthel index.

QoL assessment: The European Organization for Research and Treatment of Cancer QLQ-C30 (EORTC QLQ-C30) was used to assess QoL. The EORTC QLQ-C30 [22] comprises 30 items that cover five functional domains (physical, role, cognitive, emotional, social, and functioning), nine symptom domains (fatigue, nausea/vomiting, pain, appetite loss, diarrhea, dyspnea, constipation, sleep disturbances, and financial difficulties), and global health status. Higher scores in the functional domains and global health status indicate better health, but higher scores in the symptom domains indicate a greater symptom burden [23].

Statistical Analysis

At the start of RT and one month after RT, the stable and unstable groups were compared using the Mann-Whitney U test. In the stable and unstable groups, changes in pain, ADL, and QoL from the start of RT to one month after RT were compared using the Wilcoxon signed-rank test. Kaplan-Meier survival analysis was used to compare survival rates between the stable and unstable groups, and differences in survival curves were evaluated with the log-rank test. Statistical analyses were performed using SPSS (version 22.0, IBM Corp., Armonk, NY). All tests were two-sided, and *P*-values < 0.05 were considered significant.

## Results

Spinal instability

Patient characteristics are shown in Table 1. Compared with the start of RT, the spinal instability of eight patients from the unstable group improved one month after RT. No patients in the stable group at the start of RT worsened to the unstable group one month after RT. All patients in the unstable group used corsets for three months.

**Table 1:** Characteristics of the patients with bone metastases. ECOG PS, Eastern Cooperative Oncology Group Performance Status

Characteristic	Count (*n*)
Primary cancer site	
Lung	6
Breast	17
Prostate	10
Colorectal	8
Stomach	4
Liver	2
Pancreatic	1
Other	4
Spinal level of bone metastases	
Thoracic vertebrae	17
Lumbar vertebrae	35
ECOG PS	
0	1
1	23
2	12
3	12
4	4
Radiation therapy dose	
20 Gy	6
30 Gy	37
36 Gy	1
37.5 Gy	1
40 Gy	6
50 Gy	1

Pain

Table 2 shows the course of pain from the start to six months after RT. Pain was significantly higher in the unstable group than in the stable group at the start of RT (*P* < 0.05) and one month after RT (*P* < 0.05). Pain in the unstable group had significantly improved one month after RT compared with the start of RT (*P* < 0.05). However, pain in the stable group was not significantly different one month after RT compared to the start of RT. Pain did not worsen in any patient.

**Table 2:** Course of pain from before radiation therapy to six months after radiation therapy. Median (minimum-maximum).

	Before	1 month	2 months	3 months	4 months	5 months	6 months
Stable group	0 (0-8)	0 (0-1)	0 (0-0)	0 (0-0)	0 (0-0)	0 (0-0)	0 (0-0)
Unstable group	3 (0-10)	0 (0-3)	0 (0-3)	0 (0-2)	0 (0-1)	0 (0-0)	0 (0-0)

ADL

Table 3 shows the course of ADL from the start to six months after RT. The Barthel index at the start of RT was significantly lower in the unstable group than in the stable group (*P* < 0.05). The unstable group had a significantly improved ADL one month after RT compared to the start of RT. In the stable group, walking ability was independent in 18 patients and dependent in seven patients at the start of RT, and independent in 21 patients and dependent in 4 patients one month after RT. In the unstable group, walking ability was independent in 10 patients and dependent in 17 patients at the start of RT, and independent in 21 patients and dependent in 6 patients one month after RT. The unstable group showed a significantly improved walking ability one month after RT compared to the start of RT (*P* < 0.05).

**Table 3:** Change in activities of daily living from before radiation therapy to six months after radiation therapy. Median (minimum-maximum).

	Before	1 month	2 months	3 months	4 months	5 months	6 months
Stable group	100 (35-100)	100 (65-100)	100 (75-100)	100 (65-100)	100 (65-100)	100 (75-100)	100 (35-100)
Unstable group	75 (5-100)	100 (5-100)	100 (10-100)	100 (20-100)	100 (35-100)	100 (40-100)	100 (50-100)

QoL

The course of pain from the start of RT to six months after RT is shown in Tables 4-5. At the start of RT, physical function was significantly lower in the unstable group than in the stable group (*P* < 0.05). In the stable group, global health status, cognitive function, emotional function, pain, and sleep disturbances significantly improved one month after RT compared with the start of RT (*P* < 0.05). In the stable group, diarrhea was significantly worse one month after RT compared with the start of RT (*P* < 0.05). In the unstable group, global health status, physical function, pain, and sleep disturbances significantly improved one month after RT compared with the start of RT (*P* < 0.05).

**Table 4:** Changes in quality of life in the stable group. Median (minimum-maximum).

	Before	1 month	2 months	3 months	4 months	5 months	6 months
Global health status	33.3 (0-91.7)	50 (16.7-83.3)	50 (0-100)	50 (16.7-100)	50 (0-91.7)	50 (16.7-100)	50 (8.3-100)
Physical functioning	60 (0-100)	66.6 (6.6-100)	80 (33.3-100)	80 (26.6-100)	80 (26.6-100)	76.7 (33.3-100)	66.6 (0-100)
Role functioning	50 (0-100)	66.6 (0-100)	66.6 (0-100)	66.6 (0-100)	66.6 (0-100)	75 (0-100)	73.3 (13.3-100)
Cognitive functioning	66.6 (0-100)	83.3 (50-100)	83.3 (0-100)	83.3 (16.3-100)	83.3 (0-100)	83.3 (0-100)	75.0 (33.3-100)
Emotional functioning	58.3 (8.3-100)	83.3 (33.3-100)	83.3 (25-100)	75 (41.6-100)	83.3 (16.6-100)	83.3 (33.3-100)	79.2 (25-100)
Social functioning	50 (0-100)	66.6 (0-100)	66.6 (0-100)	66.6 (0-100)	66.6 (0-100)	66.6 (33.3-100)	83.3 (16.6-100)
Fatigue	44 (11-100)	44 (0-88)	33 (0-88)	33 (0-100)	33 (0-100)	33 (0-100)	33 (0-88)
Nausea and vomiting	0 (0-100)	0 (0-66)	0 (0-83)	0 (0-66)	0 (0-83)	0 (0-50)	0 (0-83)
Pain	66 (0-100)	33 (0-66)	33 (0-66)	33 (0-83)	33 (0-83)	33 (0-66)	24.5 (0-100)
Appetite loss	33 (0-100)	33 (0-100)	33 (0-100)	33 (0-100)	33 (0-100)	33 (0-66)	33 (0-100)
Diarrhea	0 (0-100)	0 (0-100)	0 (0-100)	0 (0-100)	0 (0-100)	0 (0-33)	0 (0-100)
Dyspnea	0 (0-100)	0 (0-66)	0 (0-100)	0 (0-33)	0 (0-100)	16.5 (0-100)	0 (0-100)
Constipation	33 (0-100)	0 (0-100)	0 (0-100)	0 (0-100)	33 (0-100)	33 (0-100)	33 (0-100)
Sleep disturbances	33 (0-100)	0 (0-66)	0 (0-100)	0 (0-66)	0 (0-100)	0 (0-100)	0 (0-100)
Financial impact	33 (0-100)	33 (0-100)	33 (0-100)	33 (0-100)	33 (0-100)	33 (0-66)	33 (0-100)

**Table 5:** Changes in quality of life in the unstable group. Median (minimum-maximum).

	Before	1 month	2 months	3 months	4 months	5 months	6 months
Global health status	33.3 (0-83.3)	50 (0-100)	50 (16.7-100)	50 (0-100)	50 (0-100)	50 (0-100)	58.3 (17-100)
Physical functioning	40 (0-80)	60 (0-93.3)	66.6 (0-100)	80 (6.6-100)	73.3 (13.3-93.3)	73.3 (6.6-93.3)	66.6 (0-100)
Role functioning	50 (0-100)	66.6 (0-100)	66.6 (0-100)	66.6 (0-100)	58.3 (0-100)	66.6 (0-100)	76.7 (6.6-86.6)
Cognitive functioning	66.6 (16.6-100)	83.3 (0-100)	66.6 (0-100)	83.3 (16.6-100)	83.3 (16.6-100)	66.5 (17-100)	75 (16.6-100)
Emotional functioning	66.6 (0-100)	66.6 (25-83.3)	75 (16.6-100)	83.3 (41.6-100)	75 (33.3-100)	83.3 (8.3-100)	83.3 (58.3-100)
Social functioning	66.6 (0-100)	66.6 (16.6-100)	66.6 (33.3-100)	66.6 (0-100)	66.6 (0-100)	66.6 (0-100)	66.6 (0-100)
Fatigue	55 (0-100)	44 (11-88)	33 (0-88)	33 (0-100)	44 (0-100)	33 (0-100)	33 (0-100)
Nausea and vomiting	0 (0-100)	0 (0-66)	0 (0-66)	0 (0-50)	0 (0-33)	0 (0-100)	0 (0-33)
Pain	66 (16-100)	66 (0-100)	66 (0-100)	66 (0-100)	66 (0-100)	66 (0-100)	66 (0-100)
Appetite loss	33 (0-100)	33 (0-100)	33 (0-100)	33 (0-100)	33 (0-100)	0 (0-100)	0 (0-100)
Diarrhea	0 (0-100)	0 (0-100)	0 (0-100)	0 (0-100)	0 (0-66)	0 (0-33)	0 (0-100)
Dyspnea	33 (0-100)	0 (0-100)	33 (0-100)	33 (0-100)	0 (0-100)	0 (0-100)	16.5 (0-100)
Constipation	33 (0-100)	33 (0-100)	33 (0-100)	0 (0-100)	0 (0-100)	33 (0-100)	0 (0-100)
Sleep disturbances	33 (0-100)	33 (0-100)	0 (0-66)	0 (0-100)	33 (0-100)	0 (0-100)	0 (0-100)
Financial impact	33 (0-100)	33 (0-100)	33 (0-66)	33 (0-100)	0 (0-66)	33 (0-100)	33 (0-100)

Prognosis

The survival rates at six months, one year, and three years were 92.0%, 64.0%, and 16.0% for the stable group and 88.9%, 55.6%, and 29.6% for the unstable group. No significant differences were found between the stable and unstable groups.

## Discussion

This study investigated the effects of wearing a corset and RT for patients with painful thoracic and lumbar bone metastases, revealing a significant reduction in pain and improved ADL and QoL in a group of people with unstable spinal stability. The group with spinal instability that was classified as stable also showed significant improvement in QoL.

Patients with spinal instability often experience increased pain during sitting and standing as a result of increased loading on the spine [24]. In some patients with spinal cord compression, prolonged bed rest can lead to complications such as depressed mood, deep vein thrombosis, chest infections, pressure sores, and urinary tract infections [25]. Wearing a brace to stabilize the spine and reduce pain by preventing or delaying vertebral collapse is an effective intervention. Exercise therapy, such as resistance training, for patients with bone metastases reduces pain and improves the strength of the paravertebral muscles [16]. In this study, no patients with spinal instability classified as unstable showed worsened pain one month after RT, and a significant improvement was found in ADL one month after RT compared with the start of RT. At the start of RT, 37% of patients showed independent walking ability, significantly improving to 77.8% one month after RT. Patients were more likely to experience lower pain while wearing a corset. Thus, patients were encouraged to move early, leading to a wider range of physical activities and improved ADL. Early mobilization also helps prevent disuse syndrome.

Research has shown that three months of RT alone is insufficient to improve QoL [26]. Rehabilitation improves functional capacity and reduces fatigue, thereby enhancing QoL over six months in patients with stable spinal metastases [27]. However, immobilization with a corset may worsen QoL in patients with bone metastases [28]. In this study, both the stable and unstable groups showed a significant improvement in QoL one month after RT compared with the start of RT. Therefore, RT and early rehabilitation were effective in improving QoL.

Successful palliation of painful bone metastasis can be achieved with RT in a time-efficient manner with very few side effects [29]. A meta-analysis revealed that >40% of patients can expect at least 50% pain relief, and fewer than 30% can expect complete pain relief within one month of RT [30]. The unstable group in this study showed significantly improved pain one month after RT compared with the start of RT. Therefore, this study supports the previous study.

Study limitations

First, the study required approximately 180 patients, but the number of patients was small, 52 patients. Second, the unstable group was difficult to compare with the corset-wearing group and the non-corset-wearing group because all patients wore corsets. Third, all participants had RT, and those who underwent surgery were excluded; hence, whether similar results could be obtained in postoperative patients remains unclear. The patients are also undergoing RT, so this study is not a validation of the corset alone. Therefore, further research is needed to examine the influences of these factors.

## Conclusions

Patients with thoracic or lumbar bone metastases who wore corsets enabled early initiation of exercise without compromising QoL. In the rehabilitation of patients with thoracic or lumbar bone metastases, wearing corsets is an intervention option, as they help reduce the load on the spine.
